# Our Advertising Department

**Published:** 1879-04-20

**Authors:** 


### *OUR ADVERTISING DEPARTMENT.*

A word to our subscribers on this subject. It is believed that THE RECORD is the best advertising medium of the kind in the Southern States. We desire to keep it so, and need the help of our readers in this matter. Let them bear in mind that it is important to know where reliable and pure medicines may be obtained, and they can order them from these advertisers, or ask their home druggist and merchants to order for them. We request and urge our readers both for their own interests, and for the good of their patients, to order from our advertisers, whom we believe to be reliable.

